# The Global Pregnancy Collaboration (CoLab) symposium on short- and long-term outcomes in offspring whose mothers had preeclampsia: A scoping review of clinical evidence

**DOI:** 10.3389/fmed.2022.984291

**Published:** 2022-08-30

**Authors:** Steven J. Korzeniewski, Elizabeth Sutton, Carlos Escudero, James M. Roberts

**Affiliations:** ^1^Department of Family Medicine and Population Health Sciences, Wayne State University School of Medicine, Detroit, MI, United States; ^2^Magee-Womens Research Institute, Pittsburgh, PA, United States; ^3^Department of Obstetrics, Gynecology and Reproductive Sciences, University of Pittsburgh, Pittsburgh, PA, United States; ^4^Group of Research and Innovation in Vascular Health, Chillán, Chile; ^5^Vascular Physiology Laboratory, Department of Basic Sciences, Faculty of Sciences, University of Bío-Bío, Chillán, Chile; ^6^Department of Obstetrics Gynecology and Reproductive Sciences, Epidemiology and Clinical and Translational Research, Magee-Womens Research Institute, University of Pittsburgh, Pittsburgh, PA, United States

**Keywords:** fetal growth restriction, intrauterine growth restriction, preterm delivery, autism, cerebral palsy, neurodevelopmental disorder

## Abstract

Preeclampsia is a maternal syndrome characterized by the new onset of hypertension after 20 weeks of gestation associated with multisystemic complications leading to high maternal and fetal/neonatal morbidity and mortality. However, sequelae of preeclampsia may extend years after pregnancy in both mothers and their children. In addition to the long-term adverse cardiovascular effects of preeclampsia in the mother, observational studies have reported elevated risk of cardiovascular, metabolic, cerebral and cognitive complications in children born from women with preeclampsia. Less clear is whether the association between maternal preeclampsia and offspring sequelae are causal, or to what degree the associations might be driven by fetal factors including impaired growth and the health of its placenta. Our discussion of these complexities in the 2018 Global Pregnancy Collaboration annual meeting prompted us to write this review. We aimed to summarize the evidence of an association between maternal preeclampsia and neurobehavioral developmental disorders in offspring in hopes of generating greater research interest in this important topic.

## Introduction

**Figure 1:**
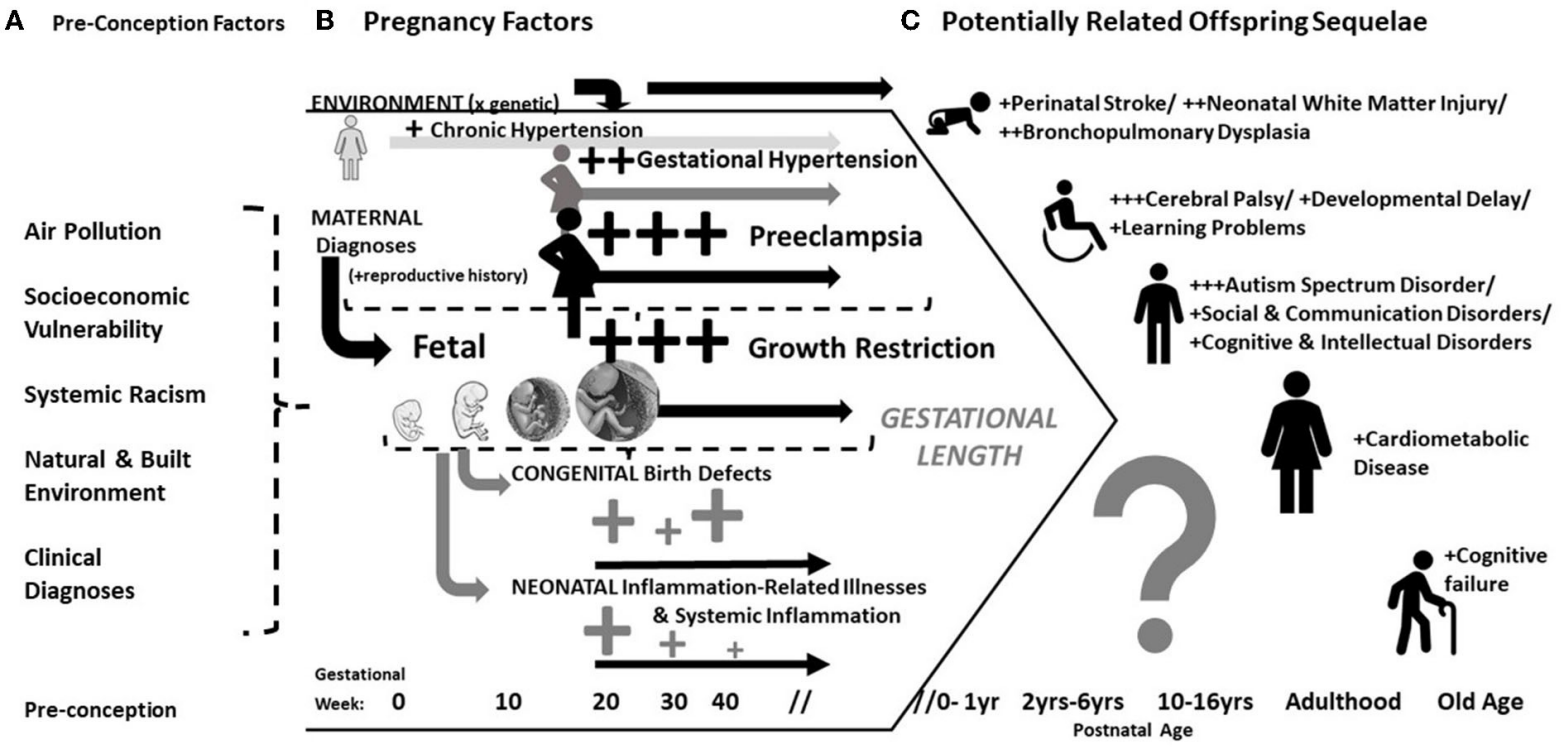
Conceptual model of the antecedents, correlates and potential sequelae in offspring of mothers with preeclampsia. **(A)** Pre-conception/environmental factors are associated hypertensive pregnancy disorders and developmental disorders; **(B)** Either by shared underlying mechanism or confounding, preeclampsia is often associated with fetal/placental diseases, congenital anomalies detected at birth, and downstream inflammation-related illnesses after birth; **(C)** Largely depending on the length of gestation (and therefore co-occurrence with fetal growth problems), preeclampsia is associated with heightened risks of multiple developmental disorders and also chronic cardiovascular disease.

It has been recognized for many years that pregnancy complications are associated with acute and chronic morbidities in offspring. Historically this has been attributed to early delivery and the failure of the infant to reach its growth potential [i.e., fetal growth restriction (FGR)]. Both conditions can occur with preeclampsia, a maternal multisystemic disorder diagnosed by new onset hypertension with other systemic involvement. Therefore, it is difficult to unravel whether preeclampsia directly effects offspring (Figure 1). This is of more than theoretical importance. Determining a direct effect of preeclampsia on offspring could provide insight into the pathogenesis of developmental disorders and hopefully inform the development of directed therapy. This review describes the historical context and modern clinical evidence of adverse outcomes in offspring whose mothers had preeclampsia or other hypertensive disorders. We conclude by discussing the challenges to discriminate an independent effect.

## Historical context of research linking pregnancy and developmental disorders

### Prematurity (a.k.a., “dysmaturity”, “immaturity” and preterm delivery) and placental dysfunction

“Prematurity” was recognized as a key risk factor for perinatal mortality and developmental disorders by mid-19th century, along with birth complications and hypoxia (1–3). The earliest definitions were imprecise [e.g., gestation beyond 6 months but before “term” (4, 5)]. The 37 week cutoff was proposed in 1902 (6), but it was not widely used until decades later (5, 7). Early obstetricians defined “dysmaturity” as a lesser amount of subcutaneous fat (1907) or weight (1915) than expected at term (8). These criteria were quickly supplanted by the more easily measured 2,500 g birth weight criterion proposed in 1919 (9). In subsequent decades a reduced birth weight for gestation was linked with developmental disability. By 1940, a review concluded that “...children born preterm demonstrate developmental retardation and that the lower the birth weight the greater the retardation (10, 11)”. Thereafter, “dysmaturity” was recognized in children born preterm and it was interpreted as a sign of “placental insufficiency” or “placental dysfunction syndrome” (8, 12, 13).

### Early suspected etiologic factors

#### Infection and inflammation

Pregnancy infections were identified as a potential cause of developmental disorders in the mid-nineteenth century. However, as early as 1895 Putnam remarked that “…*the direct effects of infection are not to be feared more than the prostrating influences of the original struggle* (p. 254)”. He proposed that infection and related processes (e.g., “edema”) can cause nervous system and cardiometabolic disorders because it “…*joins hands with developmental or inherited tendencies, or accentuate(s) the habitual strains of life*… (p. 255) (14)”. Pregnancy infection took on new meaning after 1941 when “German measles” was linked to congenital cataracts in children (15). During the 1950s−60s amniotic fluid infection syndrome was described (16) and there was speculation about microbial roots of intellectual disability (17). In 1973, evidence from autopsies prompted the inference that non-infectious inflammatory stimuli can also damage the developing brain (18). However, skepticism persisted prior to the arrival of modern microbiologic techniques.

#### Socioeconomic status, the importance of labor events and “other factors”

Social disadvantage was also implicated as an important risk factor for pregnancy and developmental disorders in children (11). In 1959, investigators surmised “…*that there are positive and probably etiologic relationships between low socioeconomic status and prenatal and paranatal (sic) abnormalities which may in turn serve as precursors to retarded behavioral development, and to certain neuropsychiatric disorders of childhood such as cerebral palsy, epilepsy, mental deficiency and behavior disorder* (19).” Social disadvantage was said to provide only a hint toward some yet unidentified mechanism. Therefore, in the late 1950s the Collaborative Perinatal Project (CPP) began to investigate: “(*1) the conditions of the pregnancy itself, such as infection, trauma, drug reactions or the progress of labor. These include normal and abnormal physiology of pregnancy, labor and delivery, (2) the environmental factors—social and economic conditions, emotional stress, or medical care—influencing parents, (3) the biological factors—age, medical and reproductive history, immunologic characteristics—in parents, and (4) the genetic background of the parents* (20)”.

#### Modern era

The CPP extended the focus of the causes of adverse outcomes for offspring from birth complications and toward intrauterine inflammation. Complexities arose as neonatal care improved in subsequent decades. As survival improved among successively earlier deliveries, postnatal morbidities were increasingly prevalent. The chronic disease era also emerged in epidemiology; with it came recognition of the implications of multicausality (21). That is, “*Cause and effect no longer seem to bear a straight lineal relationship to each other. Circular mechanisms of positive and negative feedbacks have taken over in the operational depths of homeostasis. The chain of causation is fast dissolving before our eyes to be replaced by some form of invariable association that does not lend itself readily to a graphic, mathematical, or any other representation* (22)”. Consequently, we are now tasked with determining whether preeclampsia and other pregnancy disorders are causally related to a mix of neonatal and long-term morbidities whose risk profiles overlap and often depend on gestational length. Unfortunately, while modern research has made great strides in describing underlying pathophysiology, this has not yet resulted in much progress developing interventions that substantially improve maternal and child health (23).

## Association of preeclampsia with adverse offspring outcomes

### Congenital abnormalities

#### Cardiovascular defects

Multiple studies report increased risk of birth defects with preeclampsia. However, most are not large enough to provide reliable information about specific diagnoses. In addition, criteria for preeclampsia diagnosis had changed over the years. Results also differ with early- or late-onset and pre-existing hypertension.

A large case-control study reported an elevated rate of preeclampsia in mothers of infants with cardiac defects (24). In Quebec, the risk of non-critical heart defects was elevated with preeclampsia [prevalence ratio (PR); 95% confidence interval (CI), 1.6; 1.5–1.7], after excluding stillbirths and children with chromosomal anomalies (25). Compared to late-onset, early-onset preeclampsia (<34 weeks) was associated with higher risks of both non-critical and critical heart defects (PR; 95%CI, 2.8; 1.7–4.5 and 5.6; 5–6, respectively). A Norwegian population-based study that excluded women with chronic hypertension also found children of preeclamptic pregnancies were two-fold more likely to have severe congenital heart defects than their peers whose mothers did not have preeclampsia (26). With early onset (<34 weeks), mild and severe preeclampsia were associated with two- and six-fold increased risks, respectively. By contrast, the risk of severe cardiac defects was elevated by 70% only with the severe form of late-onset preeclampsia.

At least two large studies found different risks of cardiac defects between chronic hypertension and preeclampsia superimposed onto chronic hypertension. The WHO Multi-Country Survey of 359 facilities across 29 countries reported two-fold elevated risk of cardiac defects with preeclampsia. By contrast, the risks of central nervous system, renal, limb, lip and cleft palate defects were elevated with preeclampsia superimposed onto chronic hypertension [odds ratios (ORs) > 4] (27). The Slone Birth Defects Study found four-to-six-fold increased risks of ventricular septal defects and atrial septal defects with preeclampsia superimposed on chronic hypertension (28).

#### Microcephaly

The relationship between preeclampsia or gestational hypertension and microcephaly is not clear. Some studies report an increased risk with preeclampsia (29). While the Helsinki birth cohort study found a reduced mean head circumference in children of preeclamptic mothers, children of mothers with gestational hypertension had *increased* head circumference relative to body length (30). A large extremely preterm birth cohort study found that children born severely fetal growth restricted, most of whose mothers had preeclampsia, had more than ten-fold higher risk of microcephaly than their unexposed peers (31). However, preeclampsia was not examined separately due to extensive overlap with FGR.

### Neonatal and early childhood outcomes

#### Mortality

The risk of perinatal mortality is increased with preeclampsia and severe gestational hypertension. Yet, a reduced risk was reported with mild hypertension. Gestational age and birth weight appear to moderate these relationships.

The risks of neonatal mortality (32) and infant death (33) were nearly doubled in recent studies of children of preeclamptic mothers. However, a so-called “protective” relationship was observed among the subset born preterm or with very low birth weight (34). A US population-based study of 57 million singleton live births and stillbirths at 24–46 weeks' gestation found that gestational hypertension was associated with a 30% increased risk of neonatal death. By contrast, a recent systematic review reported *reduced* odds of mortality in infants exposed to gestational hypertension (35). However, mortality risk doubled with severe hypertension and it was increased by 40% with preeclampsia based on two studies of very preterm births.

The WHO Antenatal Care Trial examined early childhood outcomes separately for preeclampsia, gestational hypertension and “unexplained FGR” (i.e., birth weight < 10th centile for gestation not associated with maternal smoking, maternal undernutrition, preeclampsia, gestational hypertension, or congenital malformations) (36). Unadjusted models showed nearly five-fold increased risk of neonatal death with preeclampsia, but no relationship with gestational hypertension. However, adjustment for study site, treatment group, socioeconomic status and non-linear birth weight resulted in non-significant associations. By contrast, adjusting only for study site and socioeconomic status, both associations were statistically significant [adjusted (a) OR; 95% CI, 4.1; 2.5–6.6 and 1.8; 1.2–2.8, respectively]. Hence, gestational age and birth weight were mediators. The risks of neonatal mortality were also increased five-fold with FGR secondary to preeclampsia and three-fold with FGR secondary to gestational hypertension, adjusting for study site, treatment group and socioeconomic status.

#### Perinatal stroke

The rate of perinatal stroke is elevated with preeclampsia, gestational hypertension and FGR. A meta-analysis of 11 studies associated preeclampsia with two-fold increased risk of perinatal stroke (37), consistent with findings from subsequent studies of term gestations (38). Larger studies reported a stronger magnitude of association. In a case-control study nested within a cohort of 199,176 births, mothers of children who suffered perinatal stroke had preeclampsia five times more often than mothers of children who without stroke. (39). In a separate multivariable model, preeclampsia and FGR were each linked with three-to-five-fold increased odds (40). Ischemic stroke also occurs more frequently in infants exposed to gestational hypertension (41).

#### Neonatal encephalopathy and white matter irregularities in children born preterm

The rate of neonatal encephalopathy, an important risk factor for perinatal stroke (42), is increased six-fold with preeclampsia (43). One study of children born extremely preterm examined maternal and fetal conditions separately. The risk of neurosonographic ventriculomegaly or echolucent lesion was nearly three-fold higher among children delivered for fetal indication without preeclampsia compared to their peers whose mothers had preeclampsia (44). Recent preliminary evidence hints at altered cerebral vessel calibers and increased regional brain volumes in children of preeclamptic mothers compared to a control group whose mothers had normal pregnancies that was matched on gestational age at delivery, sex and age at examination (45).

#### Bronchopulmonary dysplasia

BPD occurs more frequently in children of preeclamptic mothers only with preterm delivery. FGR and pre-existing hypertension seem to moderate the relationship.

The risk of BPD was increased with exposure to preeclampsia in multiple studies of different intervals of preterm delivery (e.g., <28 or <32 weeks) (46–49). By contrast, a very low birth weight cohort study reported BPD odds were decreased by 20% with preeclampsia. However, *post-hoc* analysis indicated the protective association occurred only for more physiologically mature children (50). A more recent study of 247 children born of preeclamptic pregnancies at 24– <32 weeks of gestation found four-fold increased BPD risk with preeclampsia, adjusting for nulliparity, prolonged preterm pre-labor ruptured membranes, gestational age at birth and fetal sex (51). Yet, the relationship was no longer statistically significant with additional adjustment for other intermediates: antenatal corticosteroids, birth weight Z-score, mode of delivery, respiratory distress syndrome, invasive ventilation, admission of postnatal corticosteroids, clinical or proven sepsis, treatment of a persistent ductus arteriosus.

A 2018 meta-analysis reported increased BPD risk among children born prior to 29 weeks whose mothers had gestational hypertension (35). This is consistent with an international cohort study that associated gestational hypertension with BPD risk increased by 20%, adjusting for maternal age, multiple births, sex, gestational age, birth weight z-score, antenatal steroids, mode of delivery and network (52).

In contrast to the above studies, at least one showed a protective association between chronic hypertension and BPD among births at 30–34 weeks (46). However, another study of children born at 30–34 weeks whose mothers had preeclampsia found six-fold increased BPD risk with birth weight below the 10th centile for gestation, adjusting for gestational age and other potential confounders (53).

### Neurodevelopmental and mental health disorders

#### Cerebral palsy

Preeclampsia and gestational hypertension are associated with increased CP risk, but gestational age at delivery and FGR moderate these relationships. A US study of 122,476 mother child pairs attempted to disentangle the complex relationships between preeclampsia, preterm birth and CP (54). Children of preeclamptic mothers had two-fold increased risk of CP; the earlier the diagnosis the stronger the association. However, there was no relationship between CP and term preeclampsia. By contrast, another study of children born at term associated preeclampsia with increased CP risk, but only when the child was small for gestational age (SGA) (55). A subsequent Israeli population-based study associated preeclampsia with two-fold increased risk of CP (56). Both early onset preeclampsia (<34 weeks) and SGA were “independently” linked with nine-fold and three-fold increased CP risk, respectively. However, a more recent analysis from the same regional hospital associated severe but not mild preeclampsia with elevated CP risk (57).

An Australian reconstructed total population-cohort study reported that new onset hypertension during pregnancy irrespective of proteinuria was associated with two-to-nine-fold increased risk of CP among all deliveries on each week after 27 weeks of gestation (58). A recent analysis of the Taiwan National Health Insurance Research Database further showed that a combination of preeclampsia with maternal diabetes, fetal growth restriction or preterm delivery was associated with even higher risk than preeclampsia alone (59).

#### Autism spectrum disorder

Preeclampsia and gestational hypertension are associated with a small but statistically significant increase in ASD risk. Several meta-analyses report 30%-to-50% higher ASD risk in children of mothers with preeclampsia than their unexposed peers (60–63). The relationship is not specific because chronic and gestational hypertension were each associated with similarly elevated risks (OR range 1.37–1.48) (60). Another meta-analysis showed that multivariable adjustment rendered the association between gestational hypertension and ASD non-significant; however, this should be interpreted with caution because there was no statistical difference in the relationship between ASD and preeclampsia or gestational hypertension (*p* = 0.33), and thus the justification for separating the two disorders is unclear (64). In one of the largest studies to date, combined exposure to preeclampsia and birthweight two or more standard deviations below expected was associated with higher ASD risk than preeclampsia among children with higher birthweights (65). More recently, a combined cohort study of more than four million live-born singletons in Denmark during 1978–2012 and Sweden during 1987–2010 found the strongest associations between early-/severe-preeclampsia and ASD (hazard ratios ranged from 2-to-4) (66). A recent analysis of national health insurance data additionally associated the combination of preeclampsia with maternal diabetes, fetal growth restriction or preterm delivery with even higher risk than preeclampsia alone (59). The possibly that placental dysfunction and impaired fetal growth sensitizes offspring toward even higher risks of ASD that with exposure to preeclampsia alone is further supported by a Swedish population-based study of all singleton live births (65). A separate analysis further suggests that intergenerational exposure to preeclampsia conveys heightened risk of ASD (67).

#### Schizophrenia

Preeclampsia is associated with increased risk of schizophrenia in the offspring, but FGR might mediate or moderate the relationship. A meta-analysis of 11 studies reported 40% increased risk of schizophrenia in the children of mothers with hypertensive pregnancy disorders (68). An analysis of registry data from Norway similarly reported 30% elevated odds of schizophrenia with preeclampsia (69). By contrast, a separate population-based study linked preeclampsia with more than two-fold increased risk of schizophrenia (RR; 95% CI, 2.5; 1.4–4.5), independent of congenital anomalies (70). A Danish population-based case-control study also found three-fold higher incidence of preeclampsia in mothers of children with schizophrenia than among controls, adjusting for other pregnancy complications (e.g., maternal anemia, threatened preterm delivery, hemorrhage, sepsis), family psychiatric history, urbanicity of place of birth, maternal citizenship, paternal age, and parental wealth (71). Unadjusted models also showed that lowest quintile birth weight for gestation was associated with 60% greater incidence, unlike highest quintile birth weight, when each group is compared to their middle quintile birth weight peers. However, birth weight for gestation did not convey significant risk information in the full multivariable model.

#### Attention deficit hyperactivity disorder

ADHD risk is elevated in children of women with preeclampsia, but the magnitude of association differs after multivariable adjustment for potential confounders. A recent meta-analysis of ten studies found that preeclampsia and gestational hypertension were each significantly associated with 30% increased risk of ADHD in children (64). This is consistent with a large cohort and a nested case-control study that reported significant ORs ranging from 1.2-to-1.34 for ADHD (72, 73). Stronger associations were reported in the Avon Longitudinal Study of Parents and Children. Generalized estimating equation models that adjusted for multiple sets of potential confounders consistently found approximately three-fold higher risk of ADHD in children of preeclamptic mothers compared to children not exposed (74). By contrast, a recent study of combined singleton births from Denmark and Sweden reported a smaller but significant association that was driven by two-fold elevated risk of ADHD in children whose mothers had early-/severe-preeclampsia (66). Additional evidence supports the possibility that maternal diabetes, preterm delivery or FGR might modify the association between preeclampsia and ADHD in offspring (59), although exposure continues to convey increased risk at term gestation (75). Intriguingly, intergenerational exposure to preeclampsia might play a role in ADHD was well (67).

#### Epilepsy

The incidence of epilepsy in children was increased with exposure to maternal preeclampsia at term in a large US study; by 16% with mild preeclampsia, by 68% with post term preeclampsia, by 41% with severe term preeclampsia, and by nearly three-fold with severe post term preeclampsia (76). A study of 95,450 mother-child pairs also associated preeclampsia with 60% increased risk of epilepsy only when diagnosed at term (77); a subsequent population-based cohort study of term births adds further support (75).

#### Mental health problems

Findings about the relationship between preeclampsia and offspring mental health problems are mixed (68). One cohort study linked preeclampsia with significantly increased risk of offspring psychosis (78), whereas two case-control studies found no association (79, 80). The frequency of social problems was also increased in offspring of preeclamptic mothers who were born after 34 weeks (81).

In the Helsinki birth cohort, people whose primiparous mothers had preeclampsia had higher depressive symptom scores, but this was not the case with children of multiparous women (82). Interestingly when analyzed by fetal sex, preeclampsia was associated with 55% reduced risk of mental disorder among males (83). By contrast, gestational hypertension was associated with increased risk of depression and mood disorders, but not anxiety (78, 82, 83). Gestational hypertension has also been associated with internalizing morbidity, whereas preeclampsia was linked to reduced internalizing symptoms when examined separately (84).

#### Cognitive and intellectual disorders

Preeclampsia and gestational hypertension may be associated with mild cognitive and intellectual deficits, but FGR and pre-pregnancy body mass index might moderate these relationships.

Population-based studies have reported two-fold increased odds of mild cognitive limitations in offspring of mothers with gestational hypertension, adjusting for high pre-pregnancy body mass index, multiparity, low education, and male sex (85). Similar relationships were also observed in adults. A study of 17,457 Danish conscripts found a 34% higher prevalence of low cognitive function among men whose mothers had gestational hypertension compared to their peers whose mothers were normotensive (86). Yet, the adjusted prevalence ratios were elevated with mild preeclampsia and not with severe preeclampsia or eclampsia. On the other hand, the corresponding adjusted mean IQ differences were −2.0 (95% CI, −4.0 to 0.0), −3.2 (95% CI, −4.7, −1.8), and −2.0 (95% CI, −7.2 to 3.2), respectively (86). A more recent and larger study from Norway and Sweden reported four-fold higher risk of intellectual disability in the children of mothers with early-/severe- preeclampsia compared to their unexposed peers (double the magnitude of association between any hypertensive disorder low IQ) (66).

Even at age 70 years, people whose mothers had hypertensive disorders more frequently report subjective complaints of cognitive failures (87).

Adult men in the Helsinki Birth Cohort Study whose mothers were hypertensive had slightly reduced intellectual abilities compared to their unexposed peers; parity, socioeconomic status and preterm delivery modulated these relationships (88). An Icelandic population-based study recently reported reduced mathematics scores at ages 9, 12, and 15 years in children exposed to preeclampsia or eclampsia (89). The corresponding differences were 0.44 points (95% CI, <0.01–0.89), 0.59 points (95% CI, 0.13–1.06) and 0.59 points (95% CI, 0.08–1.10), respectively, adjusting for maternal citizenship, maternal age, maternal marital status, maternal occupation, parity, singleton birth, birth year, birth place, ADHD diagnosis, place of test administration, 5-min Apgar score, SGA, gestational age at birth and whether the child was ahead or behind a grade relative to peers of the same age. By contrast, an Australian study found no relationship between gestational hypertension and offspring cognitive development when accounting for pre-pregnancy maternal body mass index (90).

Small studies have reported worse verbal and full-scale intelligence scores at 3-to-8 years in FGR children whose mothers had preeclampsia (91, 92). In contrast to these findings, at least one study found no significant differences in IQ, school achievements, or neurodevelopmental scores at age 9–10 years among fetal growth restricted children exposed to maternal hypertensive disorders (*n* = 17 with maternal preeclampsia and *n* = 25 after gestational hypertension) compared to growth restricted children of normotensive mothers (*n* = 78) (93).

### Developmental origins of adult disease

Many of the adverse outcomes recognized neonatally in infants of mothers with preeclampsia have lifelong ramifications. In addition, there are later life adverse outcomes not readily recognized at birth. Again, in many instances these have not been untangled from preterm birth and FGR.

#### Cardiometabolic and endocrine disorders

Children born at term whose mother had preeclampsia have 50%-to-60% increased incidence of endocrine, nutritional and metabolic diseases, and diseases of the blood and blood-forming organs (94). Several studies also linked preeclampsia with changes in offspring blood pressure, vascular function and body mass (95, 96). Indeed, evidence from a recent case-control study (97) supports the possibility that preeclampsia, even without co-occurring FGR, is associated with cardiac remodeling and dysfunction akin to what has been described in studies of late-onset FGR (98) in the pre- (99, 100)/neonatal (101, 102) periods and beyond (103).

A recent meta-analysis of data on 53,029 individuals in 36 studies associated preeclampsia with a ~5 mm Hg greater mean systolic, ~4 mm Hg greater mean diastolic blood pressure, and ~0.4 kg/m^2^ greater mean body mass index during childhood or young adulthood (104). There was no difference in offspring blood levels of cholesterol, triglycerides, glucose or fasting insulin. It is unclear if there are differences by subtype; e.g., a small Australian study found that only preeclampsia resulting in preterm birth was associated with three-fold increased risk of hypertension by 20 years of age (105).

An Israeli population-based cohort study of singletons that excluded mothers with chronic hypertension found a significant linear increase in the proportion of offspring who developed cardiovascular disease among women with no preeclampsia (0.24%), mild preeclampsia (0.33%), severe preeclampsia (0.51%) and eclampsia (2.73%) (106). Incidence rates were increased specifically for hypertension, arrhythmias and heart failure, unlike for cardiomyopathy and pulmonary heart disease. A similar trend was observed for offspring obesity (0.2, 0.4, 0.4, 1.4%; *p* < 0.001). Severe preeclampsia was associated with two-fold elevated risk of cardiovascular morbidity at term. However, in offspring born preterm, neither severe preeclampsia nor mild preeclampsia was associated with cardiovascular morbidity. In the long term follow up of the Helsinki study, individuals whose mothers had preeclampsia or gestational hypertension had 40%-to-90% increased risk of stroke, an association that persisted after adjustment for birthweight (30).

Several studies have found sex-differences in the relationship between preeclampsia and offspring blood pressure (107, 108). A large case-control study (145 girls and 283 boys whose mothers had preeclampsia and 12,701 girls and 20,416 boys whose mothers did not have preeclampsia) found that the risk of a systolic blood pressure > 140 mmHg at 17 years was increased by two-fold for girls, but there was no such relationship among boys adjusting for body mass index, weight and birthweight (109). Another study similarly found that adolescents exposed to preeclampsia had higher systolic blood pressure than children of normotensive mothers (115 vs. 113 mmHg, *p* = 0.03), but there was no difference in diastolic blood pressure (66 vs. 65 mmHg, *p* = 0.10); adjustment for maternal body mass and blood pressure attenuated the associations (110).

## Potential sources of variation in prior studies relating preeclampsia to adverse offspring outcomes

### Are preeclampsia and gestational hypertension different disorders?

There is overlap between preeclampsia and gestational hypertension that cannot currently be resolved. It is likely that some proportion of gestational hypertension has benign fetal outcomes. In other cases, gestational hypertension is unrecognized chronic hypertension masked by reduced blood pressure in early pregnancy (111, 112). Nonetheless, up to half of women with gestational hypertension eventually develop proteinuria or other end organ dysfunction and progression is most likely with onset prior to 32 gestational weeks (113, 114). Because gestational hypertension may raise the risk of some adverse outcomes [e.g., perinatal mortality (111, 115)], studies that fail to remove affected women from reference groups can derive spurious null associations. Early investigations tended to compare children of preeclamptic mothers to their unexposed peers whose mothers may have been hypertensive. More recently, outcomes of children whose mothers had different types of hypertensive disorders are compared to children of normotensive mothers (64). However, this is not yet universal.

### Bias in small, single-center, and referral hospital studies

Women lumped together under the banner of preeclampsia in small studies can have appreciably different characteristics that affect their progeny's risk for adverse outcomes. Investigations performed in referral hospitals are especially susceptible to bias [i.e., Berkson's bias; see (116)]. For example, a study conducted in two NICUs in Northern and Southern China reported that children of preeclamptic mothers are at three-fold increased risk of retinopathy of prematurity and multivariable adjustment for potential confounders strengthened the relationship (117). Yet, separate analyses in each hospital revealed opposite relationships, adjusting for gestational age and birth weight. Residual bias presumably from unaccounted confounders probably explains the discrepancy.

### Bias in low birth weight cohort studies

Because birth weight is tightly linked with physiologic maturation, low birth weight cohort studies tend to over-select growth restricted newborns who are more mature than their peers (118). This can distort relationships between preeclampsia and offspring outcomes that are associated with preterm delivery. For example, a *post-hoc* analysis found that a protective association between preeclampsia and BPD in a very low birth weight cohort occurred only in children who were more physiologically mature than their peers (50). Additional support comes from a study of gestational hypertension and CP performed in a low birthweight cohort and a preterm birth cohort both sampled from a Western Australian register (119). A strong protective association was observed when infants were separated by birthweight that was not present with sampling by gestational age.

Multi-center studies that enroll participants based on gestational age rather than birthweight are preferable, but this alone may not fully resolve potential bias (120, 121). Additional co-factors typically need to be considered as potential confounders. That is, the earlier the preterm delivery, the greater the number of developmental disorder risk factors. Therefore, investigators typically need to account for contributions from these other risk factors to estimate an association between preeclampsia and developmental disorders in preterm birth cohorts.

### The so-called protective relationship between preeclampsia and adverse outcomes

Protective relationships between preeclampsia and adverse offspring outcomes typically occur in studies of high-risk populations. This is probably because there is no truly “normal” or “healthy” reference group for comparison (121). Therefore, preeclampsia exposure is compared to a mixed profile of risk factors among non-preeclamptic pregnancies [e.g., intrauterine inflammation or infection (122)]. In other words, high-risk is the product of multiple exposures and preeclampsia is associated with less morbidity than others in some studies (48, 121, 123, 124). Thus, it is inappropriate to claim that preeclampsia is truly protective under such circumstances. It is more appropriate to infer that preeclampsia is associated with less risk than other pathologies in high-risk infants not exposed to preeclampsia.

### Limitations of multivariable regression

The association between preeclampsia and offspring sequelae is typically estimated using multiple logistic regression (125). These models assume no important variables are omitted, no extraneous variables are included, independent variables are measured without error, and that there are no interactions among independent variables (126). Yet, in studies of preeclampsia independent variables often violate assumptions of independence (e.g., gestational age and birth weight are correlated), measurement error is common (e.g., in gestational age estimation), and most are too small to examine interactions. Nor is it understood which variables are important or extraneous.

A major concern in recent years focuses on the pitfalls of adjusting for intermediaries (i.e., entities that lie along a causal pathway between an exposure and outcome). If unmeasured factors cause pregnancy complications and adverse offspring outcomes, then adjustment for intermediaries can bias or spuriously reverse the direction of associations (127–132). Consequently, some investigators claim that adjustment for gestational age seldom provides trustworthy answers about causation (133). Others propose a fetuses-at-risk (FAR) approach [i.e., calculating risk with all fetuses in the denominator rather than live births; (134–137)]. The FAR approach is useful for justifying indicated preterm delivery by demonstrating higher risk of stillbirth with ongoing gestation (137). However, there are important critiques when this is applied to postnatal outcomes [e.g., fetuses are unable to be diagnosed with CP, therefore FAR rates are difficult to interpret. (138–141)].

Perhaps the most cogent advice on handling gestational length in models of developmental disorders is, “…*when information about delivery-associated exposures, vulnerabilities, response modifiers, and maternal characteristics is unavailable, adjusting for gestational age at delivery (category) may be the only, if suboptimal, strategy* (p. 126)” (142). That said, judgments about causal inference in high-risk populations like extremely preterm births must consider competing risks among unexposed infants, findings among later preterm and term births and evidence from pre-clinical studies.

### Difficulties separating preeclampsia, fetal growth restriction and preterm delivery

Preeclampsia is a common indication for preterm delivery. No adjustment method can fully resolve confounding by indication in observational studies (143). Complexities arise with preeclampsia because the earlier the delivery, the more the overlap among FGR, placental malperfusion and inflammation (144–149). For example, half of all pregnancies with early-onset preeclampsia prior to 34 weeks deliver fetal growth restricted newborns (144, 146). By contrast, about three of four newborns from preeclamptic pregnancies born prior to the 28th week of gestation are fetal growth restricted (150). Indeed, recent evidence suggests that fetal growth is impaired only in severe preeclampsia and not in mild pregnancy-associated hypertensive disorders compared to normotensive pregnancy (151). Thus, to examine sequelae of FGR separate from those of preeclampsia at periviable gestational lengths requires massive sample size. Alternatively, case-control studies have contrasted preeclampsia and FGR without preeclampsia. However, if the design fails to account for gestational length differences, then preeclampsia with growth restriction and early preterm delivery is typically compared to FGR without preeclampsia and later delivery (152).

## Conclusion

Very large, carefully designed, multi-center longitudinal studies with high quality data and follow-up far beyond birth hospital discharge are needed to determine whether preeclampsia causes developmental disorders in offspring. These studies will need to consider the complex interrelationships among preconceptual and early pregnancy factors (e.g., social disadvantage, infection, obesity), fetal and placental characteristics (e.g., sex, malperfusion), maternal factors (e.g., weight gain, substance use), birth and postnatal exposures (e.g., illnesses). Iterative integration of evidence from observational studies and pre-clinical models is probably necessary to make progress considering the complexity (153, 154). If such a discrimination is achieved, then emerging data on the pathogenesis of preeclampsia can inform relevant pathways for adverse fetal outcomes and perhaps guide interventions.

## Author contributions

SK conceived the writing project, wrote the first draft of the manuscript, critically reviewed/revised iterations, and approved the final submission. ES, CE, and JR critically reviewed/revised iterations of the manuscript and approved the submitted version. All authors contributed to the article and approved the submitted version.

## Funding

CE was funded by Fondecyt 1200250 (Chile).

## Conflict of interest

The authors declare that the research was conducted in the absence of any commercial or financial relationships that could be construed as a potential conflict of interest.

## Publisher's note

All claims expressed in this article are solely those of the authors and do not necessarily represent those of their affiliated organizations, or those of the publisher, the editors and the reviewers. Any product that may be evaluated in this article, or claim that may be made by its manufacturer, is not guaranteed or endorsed by the publisher.
